# The Effects of *Aronia melanocarpa* Extract on Testosterone-Induced Benign Prostatic Hyperplasia in Rats, and Quantitative Analysis of Major Constituents Depending on Extract Conditions

**DOI:** 10.3390/nu12061575

**Published:** 2020-05-28

**Authors:** Na-Hyun Kim, Jonghwan Jegal, Yun Na Kim, Jeong-Doo Heo, Jung-Rae Rho, Min Hye Yang, Eun Ju Jeong

**Affiliations:** 1 Gyeongnam Department of Environment & Toxicology, Korea Institute of Toxicology, 17 Jegok-gil, Munsan-eup, Jinju-si, Gyeongsangnam-do 52834, Korea; nhkim@kitox.re.kr (N.-H.K.); jdher@kitox.re.kr (J.-D.H.); 2 College of Pharmacy, Pusan National University, Busan 46241, Korea; jhjegal@pusan.ac.kr; 3 Department of Agronomy and Medicinal Plant Resources, Gyeongnam National University of Science and Technology, Jinju 52725, Korea; skdbssk@hanmail.net; 4 Department of Oceanography, Kunsan National University, Kunsan 54150, Korea; jrrho@kunsan.ac.kr

**Keywords:** *Aronia melanocarpa*, benign prostatic hyperplasia, 5-alpha-reductase, testosterone, androgen receptor, constituents

## Abstract

This study aimed to investigate the beneficial effects of *A. melanocarpa* on testosterone propionate (TP)-induced benign prostatic hyperplasia (BPH) in Wistar rats. Moreover, the bioactive constituents in the extract were determined using LC/MS and HPLC analyses. The dried fruits of *A. melanocarpa* were extracted using accelerated solvent extraction (ASE) under different extract conditions (temperature, 30 °C or 100 °C; extract solvent, 60% or 100% ethanol) to yield four extracts (T1~T4). Of the four *A. melanocarpa* extracts, T1 extracted under the condition of 100% ethanol/low temperature (30 °C) exhibited the greatest inhibitory activity on TP-induced prostatic hyperplasia in rats. The administration of T1 (100 mg/kg body weight, p.o.) for six weeks attenuated TP-induced prostate enlargement and reduced the levels of dihydrotestosterone (DHT) and 5α-reductase in both serum and prostate tissue. The suppression of PCNA mRNA expression in prostate tissue was remarkable in T1-treated rats. In LC/MS analysis, the levels of main anthocyanins and phenolics were significantly higher in T1 than in the other extracts. Furthermore, the quantitative study showed that the contents of cyanidin-3-glucose and cyanidin-3-xylose in T1 exhibited 1.27~1.67 and 1.10~1.26 folds higher compared to those in the other extracts. These findings demonstrated that *A. melanocarpa* extract containing anthocyanins as bioactive constituents attenuated the development of testosterone-induced prostatic hyperplasia, and suggested that this extract has therapeutic potential to treat prostate enlargement and BPH.

## 1. Introduction

Benign prostatic hyperplasia (BPH) is a pathologic process that is more likely to develop after middle age [1]. More than 50% of men aged over 60 have BPH and 15% to 30% of those affected suffer lower urinary tract symptoms, such as urinary retention, bladder infection, bladder calculi, or renal failure [2,3]. These complications not only impair quality of life but may lead to the need for medicinal treatment or surgical interventions like prostatectomy and its attendant risks of morbidity and death [2]. Patients with BPH characteristically have increased numbers of epithelial and stromal cells in prostate tissues [4]. The precise mechanism responsible for the development of hyperplasia has not been definitively established, but sex hormones, stromal-epithelial interaction, growth hormones, and neurotransmitters acting in concert may result in abnormal cell death or apoptosis or the excessive proliferation of epithelial and stromal cells which give to the pathogenesis of BPH [5,6].

BPH is known to depend on androgens, especially dihydrotestosterone (DHT) [7], which is the predominant hormone in immature, mature, and hypertrophic prostate glands [7,8]. DHT is synthesized from testosterone by two isoenzymes of 5α-reductase, that is, types 1 and 2 [9], and it is known that the effects of testosterone in reproductive organs are amplified by the local conversion of testosterone to DHT [9,10]. Type 2 5α-reductase is found predominantly in prostate and other genital tissues, whereas type 1 is found throughout the body where 5α-reductase is expressed, including the skin, liver, and prostate [11]. Furthermore, the therapeutic effects of 5α-reductase inhibitors, such as finasteride and dutasteride, on prostate hyperplasia have been firmly established, and their adverse event profiles make them suitable for long-term use [11,12].

*Aronia melanocarpa* (Michx.) Elliot, commonly referred to as chokeberry, is a member of the Rosaceae family and was introduced to Eastern Europe from North America [13]. *A. melanocarpa* has been reported to exhibit various biological activities, such as hepatoprotective [14], hypolipidemic [15], and cardiovascular-protective effects [16]. The fruits of *A. melanocarpa* have been traditionally used as a natural remedy by Potawatomi Native Americans to treat colds. This medicinal effect of chokeberries may be due to their antimicrobial activities. In various screening experiments for antimicrobial activity, it was found that the chokeberry prevented biofilm formation and inhibited bacterial growth. Handeland et al. (2014) reported that the intake of chokeberry juice causes a strong antibacterial effect in people suffering from urinary tract infections [17]. For six months, chokeberry juice containing a high content of total phenolics including B-type procyanidins, anthocyanins, and chlorogenic acids were applied to residents in nursing homes. The results revealed that the significant reduction in antibiotics toward urinary tract infection was observed during the period of juice administration. Regarding the beneficial effects of chokeberries for urinary system disease, Kirakosyan et al. (2015) have noticed that chokeberry anthocyanins are distributed mainly into the urinary bladder and the kidney after oral intake in rats [18].

The fruits of *A. melanocarpa* are rich in phenolic compound and mainly include four cyanidin glycosides: 3-galactoside, 3-glucoside, 3-arabinoside, and 3-xyloside [19]. Procyanidins and anthocyanins are responsible for the astringent taste and dark violet color, respectively, of its berries [20]. These anthocyanins exhibit the beneficial effects of the fruit on cancer and cardiovascular diseases [21]. However, there is still a limitation in the commercial use of natural resources containing anthocyanins due to their high reactivity that is vulnerable to temperature, pH value, water activity, and oxygen, etc. Anthocyanins are readily degraded and structurally converted under the undesirable conditions [22,23,24,25,26,27]. The presence of co-pigments, light, concentration, metallic ions, enzymes, sugars, and proteins are reported to affect the color and stability of anthocyanins [28,29,30].

Based on the traditional use of chokeberries and the biological activities recently reported, in the present study, we attempted to evaluate the potential of chokeberries to improve a urinary system-related disease—BPH. Moreover, we aimed to determine whether anthocyanin, a major bioactive component in chokeberries, contributes to the therapeutic effects on BPH in rats. Four ethanolic extracts of *A. melanocarpa* fruits were prepared using a different temperature and extraction solvent, which affects the stability and yield of anthocyanins contained in *A. melanocarpa*. The effects of four extracts on prostate enlargement, the protein expressions of DHT and 5α-reductase (5AR), the mRNA expressions of androgen receptors (AR), prostate-specific antigen (PSA), and proliferating cell nuclear antigens (PCNA) in prostate tissues were evaluated in a testosterone propionate (TP)-induced BPH rat model. The contents of anthocyanins and phenolics in four *A. melanocarpa* extracts were analyzed using LC/MS and HPLC experiments.

## 2. Materials and Methods

### 2.1. Sample Preparation

The fruits of *A. melanocarpa* (Michx.) Elliot were obtained from the Samheung Agricultural Corporation (Geochang, South Korea) and identified by Professor Yang, Min Hye (College of Pharmacy, Pusan National University). A voucher specimen (GNP-78) has been deposited in the Laboratory of Pharmacognosy, College of Life Sciences, Gyeongnam National University of Science and Technology.

### 2.2. A. Melanocarpa Extraction

Accelerated solvent extraction (ASE) was performed using a BUCHI Speed Extractor E-916 (BUCHI Labortechnik AG, Swiss). The fruits of *A. melanocarpa* were freeze-dried for 5 days and cut into small pieces prior to extraction, which was performed as follows. Two grams of a mixture of 0.5 g of Aronia powder and 1.5 g of Celite was loaded into a 20 mL PLE cell and the residual volume was filled with quartz sand. The extraction conditions used were: extraction temperature (30 °C or 100 °C), solvents (60% or 100% ethanol), one extraction cycle, hold time (10 min), and extraction pressure (100 bar). Each extract (20 mL) was collected in a 60 mL glass vial, and left to rest in air for 15–20 min. For the in vivo study, the extract obtained was concentrated in vacuo and was then freeze-dried to remove extract solvent. The powdered extract was solved and diluted with 0.5% sodium carboxymethyl cellulose (CMC-Na) and administered to the animals. For chemical study, the extract obtained by ASE was diluted with MeOH and directly injected into HPLC or LC/MS after being filtered through a 0.45 um nylon membrane.

### 2.3. LC/MS Analysis of Aronia Extract

Relative amounts of anthocyanins and polyphenols in Aronia extract were determined by Multiple Reaction Monitoring (MRM). The system used for analysis consisted of an Agilent 1200 HPLC unit and an ABSCIEX QTRAP 3200 mass spectrometer. For chromatographic separation, 5 uL of Aronia extract prepared using ASE was directly injected into a Synergi Fusion column (2.6 um, 2.1 × 50 mm) at a flow rate of 0.35 mL/min under a gradient elution using solvents A (0.1% H_2_O) and B (0.1% methanol) using the following program; 15%–50% B from 0~5 min, 50%–100% B from 5 to 6 min, and 100% B from 6 to 7.5 min, followed by 4 min equilibration with 15% B. Three anthocyanins were identified in positive MRM mode (449/287 for cyanidin-3-galactoside or cyaniding-3-glucoside, 419/287 for cyanidin-3-arabinose, and 287/287 for cyanidin), and two phenols were identified in negative mode (353/191 for caffeonylquinic acid and 595/301 for quercetin vicianoside).

### 2.4. High Performance Liquid Chromatography Analysis of Phenolic Compounds

Quantification of phenolic compounds in the ASE extracts were determined with high performance liquid chromatography (HPLC). The system consists of a Thermo Dionex Ultimate 3000 HPLC system with a pump, an auto sampler, a column compartment, and a diode array detector (DAD-3000). Aronia extract prepared using ASE was filtered through a 0.45 um nylon membrane filter and diluted with Methanol (1:1) were injected into the HPLC. The column (Phenomenex Gemini, 4.6 mm × 250 mm) and sampler temperatures were maintained at 30 and 10 °C, respectively. Solvent A (0.1% trifluoroacetic acid in water) and solvent B (acetonitrile) were used as the mobile phases (0–5 min, 10%–15% B; 5–15min, 15% B; 15–45 min, 15%–30% B). The flow rate was 1.0 mL/min. Injection volume was 10 uL and detection was performed at 280 nm.

### 2.5. Animals

A total of 42 Male Wistar rats (7 weeks old) of an average body weight of 250 ± 10 g were purchased from Orient Bio (Seoul, South Korea). Animals were acclimatized for two weeks under a 12 h:12 h light-dark cycle at 20 ± 2 °C and 50 ± 5% room humidity with ad lib access to food and water. Animal experiments were carried out according to the guidelines issued by the Gyeongnam Department of Environment & Toxicology, Korea Institute of Toxicology on the Care and Use of Laboratory Animals. The animal care and protocol was reviewed and approved by the IACUC (Institutional Animal Care and Use Committee) at the Korea Institute of Toxicology Gyeongnam Department of Environmental Toxicology and Chemistry (approval No. 1609-0004).

### 2.6. Induction of BPH and Treatment

To exclude the influence of testosterone, 36 rats were castrated by removing the testes and epididymis. The remaining 6 rats were given the sham surgery. For castration surgery, animals were anesthetized by intramuscular injection of Zoletile 20 mg/kg + Rompun 10 mg/kg body weight. Preoperative antibiotics were administered subcutaneously (Cephazolin 20 mg/kg). One week after the surgical operation, test materials were orally treated and prostatic hyperplasia was induced by injecting 3 mg/kg testosterone propionate (TP, Tianjin Jinyao Amino Acid Co. Ltd.; batch no. 1301141) subcutaneously daily for 6 weeks. Castrated animals were randomly allocated to six groups (*n* = 6, per group), as detailed below. During the 6 week prostatic hyperplasia induction period, the animals in the sham group and the VC group received 0.5% CMC-Na. The animals in the positive control group were administered with TP subcutaneously and given saw palmetto by oral gavage at a dose of 100 mg/kg body weight daily, and animals in the four test groups (T1~T4) were administered TP subcutaneously and treated with Aronia extract at the dose of 100 mg/kg body weight daily for 6 weeks. Summarizing:

1. Group Sham: non-BPH-induced and received oral 0.5% CMC-Na
2. Group VC: BPH-induced and received oral 0.5% CMC-Na
3. Group PC: BPH-induced and received saw palmetto (100 mg/kg body weight, oral gavage)
4. Group T1: BPH-induced and received 100% ethanol/low temperature (30 °C) *A. melanocarpa* extract (100 mg/kg body weight, oral gavage).
5. Group T2: BPH-induced and received 100% ethanol/high temperature (100 °C) *A. melanocarpa* extract (100 mg/kg body weight, oral gavage).
6. Group T3: BPH-induced and received 60% ethanol/low temperature (30 °C) *A. melanocarpa* extract (100 mg/kg body weight, oral gavage).
7. Group T4: BPH-induced and received 60% ethanol/high temperature (100 °C) extracted *A. melanocarpa* (100 mg/kg body weight, oral gavage).

### 2.7. Tissue, Blood Collection, and Biochemical Analysis

After a treatment period of 6 weeks, rats were sacrificed by CO_2_ chamber and a blood sample was obtained from the abdominal aorta. Ventral prostate was collected and weighed before being frozen. Blood samples were left in separation tubes at room temperature for 30 min, and centrifuged at 3000 rpm for 15 min. Serum levels of total protein, albumin (ALB), blood urea nitrogen (BUN), creatinine (CREA), aspartate transaminase (AST) alanine transaminase (ALT), alkaline phosphatase (ALP) total bilirubin (Bil), triglyceride (TG), and total cholesterol (TCHO) were measured using a Hitachi 7180 Automatic Analyzer (Hitachi High Technologies, Seoul, South Korea).

### 2.8. Real-Time Reverse Transcriptase Polymerase Chain Reaction

Total RNA extraction and cDNA synthesis from the rat prostate tissue were followed using the protocols of the manufacturer (Qiazol lysis reagent, QuantiTect reverse transcription kit; QIAGEN, Hilden, Germany). Real-time PCR based on SYBR-green step was performed by Stratagene Mx3005P thermocycler (Agilent technologies, Inc., Santa Clara, CA, USA) and PCR conditions were as follows: 95 °C for 2 min, then 40 cycle of 95 °C for 15 s and 60 °C for 1 min, according to the user manual of Gotaq qPCR master mix (Promega Corporation, Madison, WI, USA). All samples were run in triplicate and were analyzed by the ΔΔCt method. Moreover, each datum was normalized by GAPDH mRNA level. Primer sequences are listed as follows; AR (forward; gggtgacttctctgcctctg, reverse;ccggagtagttctccatcca), PSA (forward;gggggcaaagatatatgcaa, reverse;gcacaccatcacaaatgagg), PCNA (forward;ttggaatcccagaacaggag, reverse;agaaaacttcac cccgtcct), GAPDH (forward;agacagccgcatcttcttgt, reverse;tgatggcaacaatgtccact).

### 2.9. Prostate Index (PI) and 5AR and DHT Expressions in Prostate Tissues

Body weight and prostate size were accurately measured after a treatment period of 6 weeks. To determine the levels of 5AR and DHT in prostate tissue, the entire prostate was homogenized in protein lysis buffer. After centrifuging at 12,000 rpm, the protein was extracted and the concentration was determined using a Bradford protein assay. The levels of 5AR and DHT in protein extract were quantified using ELISA kits (Cusabio Biotech Co., LTD. #CSB-EL022654RA for 5AR; MyBiosource MBS265478 for DHT).

### 2.10. Statistical Analysis

All statistical analysis was performed with SPSS statistics 17.0 program (SPSS Inc., IL, USA), one-way ANOVA followed by Dunnett’s post hoc test which was used for the data satisfied with Levene’s test. Otherwise, a non-parametric Kruskal–Wallis test with Dunn’s test was performed. *p* values of <0.05 were considered as significant. The results were converted to the graph by GraphPad Prism 5 (CA, USA), in which all columns of the graph had error bars that denoted the standard error of the mean.

## 3. Results and Discussion

### 3.1. Effects of A. Melanocarpa Extracts on PI and Serum Liver Enzymes in the TP-Induced BPH Murine Model

**Figure 1:**
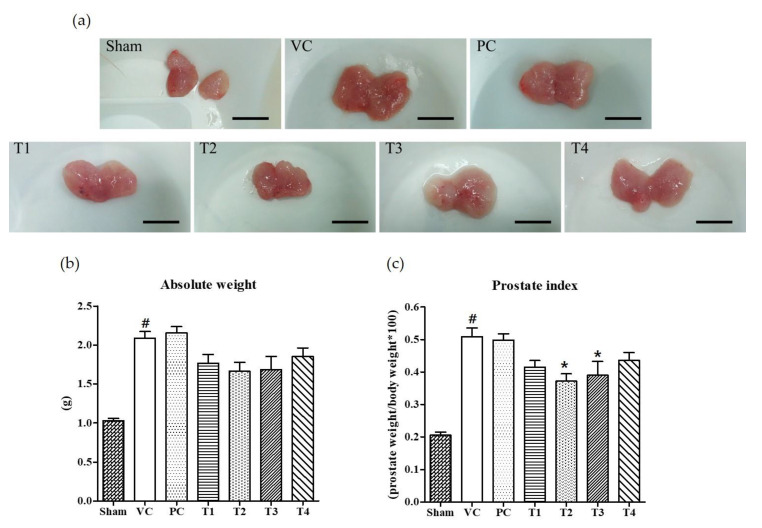
Effects of orally administered *A. melanocarpa* extracts, T1~T4 on prostate weights and prostate indices of testosterone propionate-treated Wistar rats. Clinical features of TP-induced prostate hyperplasia; Bar = 10 mm (**a**), absolute prostate weights (**b**), and prostate indices (**c**). Results were expressed as the mean value (± SD) of animals in each group. ^#^ *p* < 0.001 vs. the sham group; * *p* < 0.05 vs. the VC group. Sham, non-BPH-induced and received 0.5% CMC-Na; VC, BPH-induced and received 0.5% CMC-Na; PC, BPH-induced and saw palmetto-treated group. The preparation and administration of T1~T4 are described in Materials and Methods.

Six weeks after the induction of BPH by TP injection and the co-treatment of test samples into seven-week-old Wistar rats, it was found that the absolute prostate weight and PI was considerably increased as compared to non-TP-treated controls (Sham group) (Figure 1). Commercial saw palmetto product (PC group), 100% ethanol/low-temperature (T1), 100% ethanol/high-temperature (T2), 60% ethanol/low-temperature (T3), and 60% ethanol/high-temperature (T4) extracts of *A. melanocarpa* fruits were tested for their protective effects on TP-induced prostatic hyperplasia in rats. As shown in Figure 1b, the average weight of prostate and PI was lower in rats treated with *A. melanocarpa* extracts compared to that of TP-treated vehicle controls (VC group). Of the four *A. melanocarpa* extract groups (T1~T4), the administration of T2 produced the greatest reduction in the PI (Figure 1c), which was calculated by expressing prostate weight as a percentage of body weight. PI has been proposed to be a risk factor of BPH and prostate cancer (Rodriguez et al., 2001). Saw palmetto (*Serenoa repens*) is commonly recommended as an alternative to drugs for treating lower urinary tract symptoms (LUTS) attributed to BPH [31]. However, in the present study, no significant therapeutic effect on prostate enlargement was observed in rats treated with saw palmetto for six weeks at a dose of 100 mg/kg body weight as a positive control (PC). The absolute weight of prostate and PIs tended to be lower in all *A. melanocarpa*-treated groups (T1~T4) compared to the VC group, whereas statistical significance was found only in T2- or T3-treated groups.

Additionally, we checked serum chemistry for screening the overall clinical status of rats (Table 1). All biochemical indexes except some liver enzymes were in the normal range and no significant differences between the groups were found. Interestingly, serum AST values decreased in all *A. melanocarpa*-treated groups (T1~T4), and ALP values decreased in T4 group. Though the changed activities of liver enzymes by *A. melanocarpa* are not directly related to the beneficial effects on BPH, it is a favorable outcome for functional food development using *A. melanocarpa*.

### 3.2. Effects of A. Melanocarpa Extracts on the Expression Levels of DHT and 5AR in Serum and Prostate Tissues

**Figure 2:**
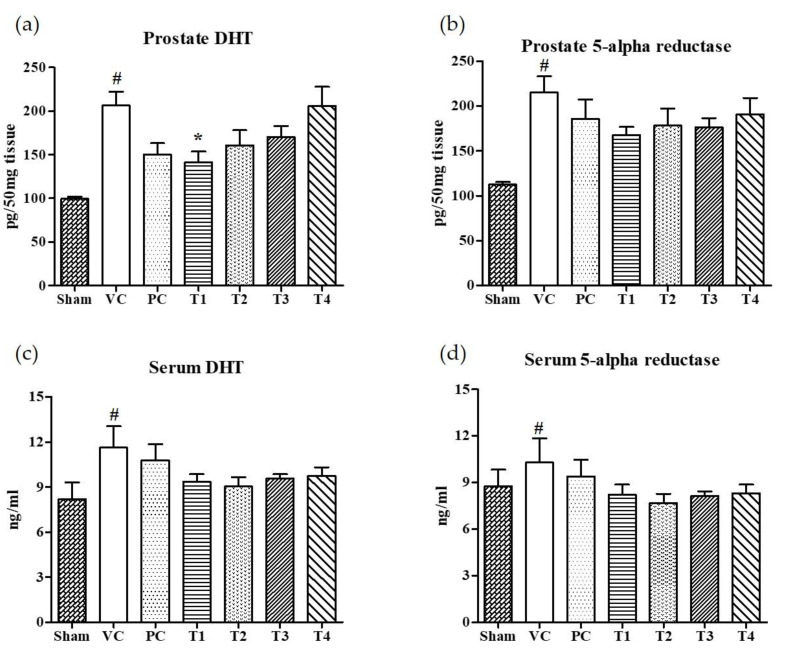
Effects of orally administered *A. melanocarpa* extracts T1~T4 on dihydrotestosterone (DHT) and 5α-reductase levels in prostate tissues and sera of testosterone propionate-treated Wistar rats. DHT levels in prostate (**a**), 5α-reductase levels in prostate (**b**), DHT levels in serum (**c**), and 5α-reductase levels in serum (**d**). Results were expressed as the mean value (± SD) of animals in each group. ^#^ *p* < 0.01 vs. the sham group; * *p* < 0.05 vs. the VC group. The preparation and administration of T1~T4 are described in Materials and Methods.

The 5α-reductase-mediated conversion of testosterone to dihydrotestosterone (DHT) in androgen responsive target cells is considered to be an obligatory step for the development of BPH [32]. Moreover, the inhibitors for 5AR can prevent and retard the progression of BPH by suppressing DHT synthesis [32,33]. Finasteride, 4-aza-steroid, is a selective inhibitor for type 2-5AR which reduces the level of serum DHT [34]. However, men who took finasteride for urinary problems experienced side effects including an increased risk of reduced libido, impotence, and erectile dysfunction [35]. Recently, BPH patients have turned to the use of natural products to find a safer and more effective means of treating BPH [36,37]. In the present study, the effects of four *A. melanocarpa* extracts (T1–T4) on the levels of DHT and 5AR in both serum and prostate tissues were evaluated. Of the four extracts, T1 showed the greatest inhibition on DHT levels in prostate tissue (Figure 2a) compared to the VC group (Figure 2b). Though no statistical significance was found, the relative reduction in DHT and 5AR levels in both serum and prostate tissue were found in all *A. melanocarpa* extract-treated groups (T1–T4). Saw palmetto is well known to decrease prostate DHT and 5AR, and attenuate testosterone-induced prostate growth and hyperplasia in vivo [38,39,40]. Furthermore, it has been reported that Saw palmetto decreased prostate-specific antigen (PSA) levels in men with enlarged prostates [41]. Now, a variety of supplements containing Saw palmetto extract are commonly consumed by men with prostate enlargement and BPH. In our experiment system, we observed the tendency of DHT and 5AR levels to decrease in serum and prostate tissue in the Saw palmetto-treated group (PC), however, no statistical significance was found when compared to the VC group.

### 3.3. Effects of A. Melanocarpa Extracts on the mRNA Expressions of AR, PSA, and PCNA

**Figure 3:**
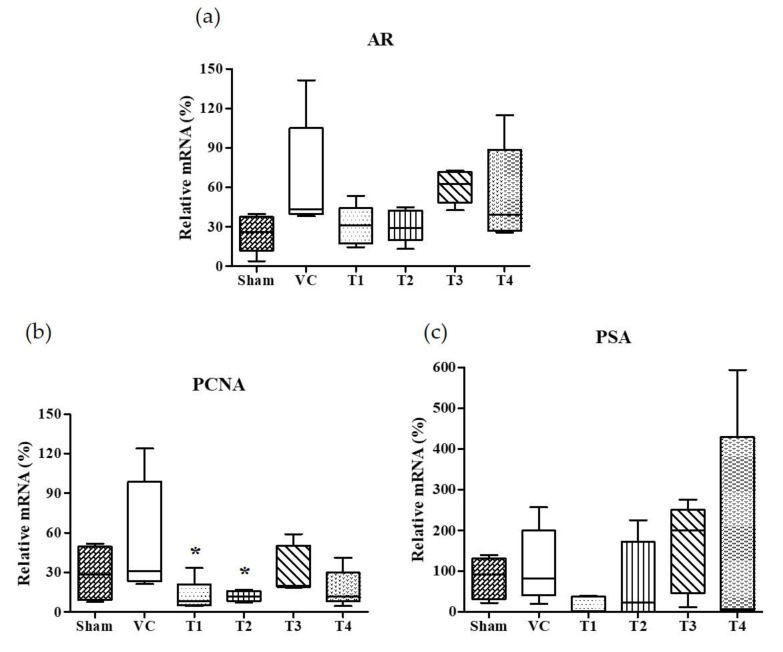
Effects of orally administered *A. melanocarpa* extracts T1~T4 on androgen receptor (AR), prostate specific antigen (PSA), and proliferating cell nuclear antigen (PCNA) protein expressions in the ventral prostate tissues of testosterone propionate-treated Wistar rats. AR mRNA expressions (**a**), PSA mRNA expressions (**b**), and PCNA mRNA expressions (**c**). Results were expressed as the mean value (± SD) of animals in each groups. * *p* < 0.05 vs. the VC group. The preparation and administration of T1~T4 are described in Materials and Methods.

Androgens and AR, a member of the steroid receptor superfamily, are essential for prostate development, growth, and function [42]. In androgen-responsive prostate cells, AR is required for the initiation of androgen-dependent gene transcription and plays an important role in the progression of hormone-refractory prostate cancer [43,44,45]. The activated AR by androgen is dissociated from chaperones and translocated to nuclei. It has been known that the binding of AR with androgen response elements (AREs) induce the gene expression of PSA and PCNA [46,47]. PSA, a key androgen-regulated gene, is sensitive and is a selective marker of prostate cancer, and as such is used for screening and assessment purposes [48]. Accordingly, we examined the effects of the four *A. melanocarpa* extracts on AR and androgen-regulated genes, PCNA and PSA expressions in prostate tissues. As shown in Figure 3, the standard deviation of AR, PCNA, and PSA in the VC group was considerably large. Though we observed the tendency of AR and PSA mRNA expressions to decrease in *A. melanocarpa*-treated groups, the statistical significance of this was not obtained. Based on the mean value of each group, the administration of T1 and T2 reduced the mRNA expression of AR to the level of 46.3% and 46.2%, respectively (T1: 30.85 ± 15.13, T2: 30.75 ± 12.36) of the VC group (66.55 ± 43.60) (Figure 3a). In addition, the mRNA expression of PSA was inhibited to 13.6% and 65.2% of the VC group (112.7 ± 91.77) by the administration of T1 and T2, respectively (T1: 15.36 ± 20.27, T2: 73.53 ± 97.82). The significant change on mRNA expression of AR, PCNA, and PSA were not observed in either the T3- or the T4-treated group. We found that the administration of T1 showed a tendency to reduce the TP-induced mRNA overexpression of AR, PSA, and PCNA in the prostate. As for the T2-treated group, the reduction in AR and PCNA expressions were similar to the T1-treated group, while a reduction in PSA expression was not observed. Taken together, T1 and T2 samples are highly effective at improving TP-induced BPH in rats, although some markers showed inconsistent results (T1; PI index, T2; prostatic DHT, and PSA level) and leave some questions about its underlying mechanisms. It needs further research including transmission electron microscopy to confirm prostatic cell morphology.

### 3.4. Comparison of Phenolic Compounds Abundance in A. Melanocarpa Extracts by LC/MS Analysis

**Figure 4:**
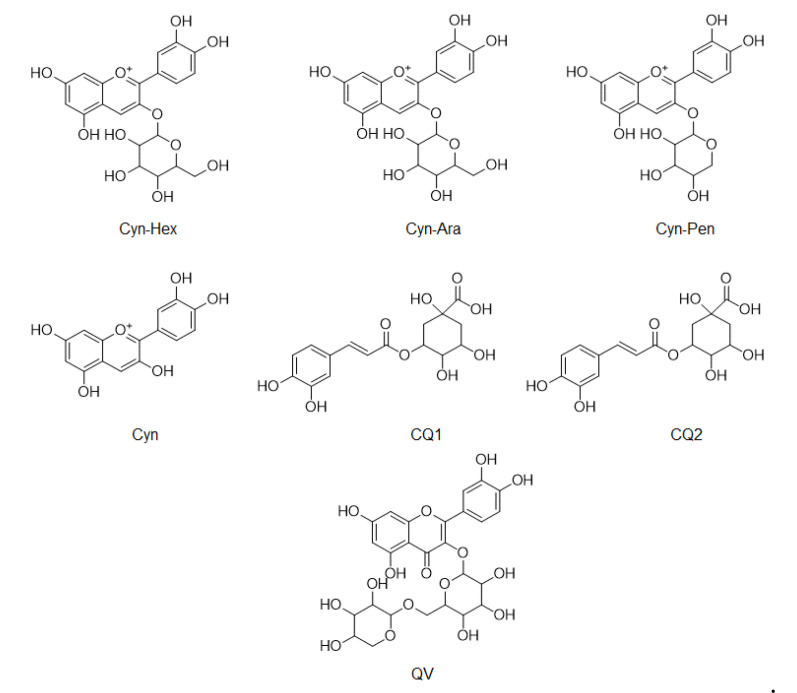
Chemical structures of compounds detected in LC/MS

LC/MS was used to compare the compositions of phenolic compounds in the four extracts of *A. melanocarpa* (Table 2). The abundance of anthocyanins and phenols reported as mainly being contained in *A. melanocarpa* was detected using LC/MS in Multiple Reaction Monitoring (MRM) mode. Figure 4 shows the chemical structures of the seven compounds detected in T1~T4, namely, Cyn-Hex (cyanidin-3-glucose or cyanidin-3-galactose), Cyn-Ara (cyanidin-3-arabinose), Cyn-Pen (cyanidin-3-pentose), Cyn (cyanidin), CQ1 (caffeonylquinic acid), CQ2 (caffeonylquinic acid), and QV (quercetin vicianoside). As a result, the levels of all seven compounds were highest in T1. The relative abundance of the main anthocyanin in *A. melanocarpa*, Cyn-Hex (cyanidin-3-glucose or cyanidin-3-galactose) was about 2.5 fold times that of T4.

### 3.5. Quantification of Main Anthocyanin and Phenolics in A. Melanocarpa Extracts Using HPLC

**Figure 5:**
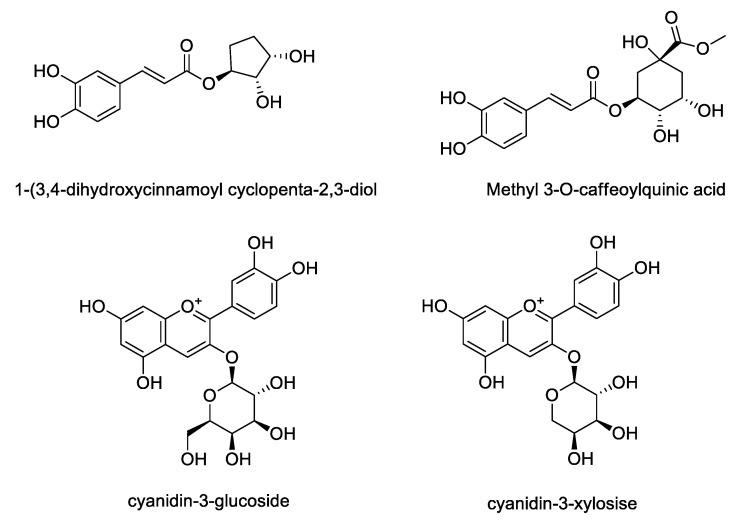
Chemical structures of compounds isolated from *A. melanocarpa* fruits

Based on LC/MS analysis, the content of four phenolic compounds in *A. melanocarpa* extract was quantified using HPLC. Though chromatographic separation, four compounds including 1-(3, 4-dihydroxycinnamoyl cyclopenta-2, 3-dilo), methyl 3-O-caffeoylquinic acid, cyanidin-3-glucoside and cyanidin-3-xyloside were isolated from the extract of *A. melanocarpa* fruits (Figure 5). As shown in Table 3, three compounds other than methyl 3-O-caffeoylquinic acid were detected in *A. melanocarpa* extract using HPLC. The contents of these compounds detected in *A. melanocarpa* extract T1~T4 were quantified based on a comparison of the retention time and the UV spectrum with standard compounds. The significant difference between groups were found in the content of cyanidin glycosides. The contents of cyanidin-3-glucose (0.7642 mg/mL) and cyanidin-3-xylose (2.6843 mg/mL) were highest in T1. The content of cyanidin-3-glucose in T1 was shown to be 1.27–1.69 fold higher than the other extracts (T2~T4).

The stability of anthocyanin is readily affected by temperature, pH value, and water activity during preparation and storage. It is known that water solubility is essential in degradation of anthocyanins [24]. Hence, decomposition index (DI) and water activity are recognized as major indicators when evaluating the stability of anthocyanins [27]. In this study, ethanol, one of the most commonly used solvents in commercial processes, was selected as the extraction solvent. Four extraction conditions were applied by varying the temperature (30 °C and 100 °C) and water content which affects the stability of anthocyanins. As a result, we observed that two main anthocyanins of *A. melanocarpa*, cyanidin-3-glucoside, and cyanidin-3-xyloside were stably extracted with ethanol without water under low temperature conditions (T1). From emerging evidence regarding the beneficial effects of polyphenols commonly found in fruits and vegetables on the prevention and treatment of prostate cancer and BPH [49,50], we supposed that the abundant components of polyphenols mainly including anthocyanins in T1 might have contributed to its beneficial effects on BPH in our TP-induced rat model.

## 4. Conclusions

In the TP-induced BPH murine model, the administration of the four *A. melanocarpa* extracts (T1~T4) tended to lower the absolute weight of the prostate and PI. Of the four extracts, T1 showed the potent inhibition on DHT level in prostate tissue induced by TP. Moreover, the mRNA expression of PCNA in the prostate were reduced in the T1-treated group. In LC/MS, the relative abundance of the main anthocyanins and phenolics contained in *A. melanocarpa* were highest in T1 compared to the other extracts. The quantitative study using HPLC showed that the contents of cyanidin-3-glucose and cyaniding-3-xylose are highest in T1. Accordingly, our findings suggest that T1, the extract of *A. melanocarpa* fruits extracted under the condition of 100% ethanol/low temperatures (30 °C) effectively inhibited the development of TP-induced prostatic hyperplasia, and the anthocyanin glycosides are thought to contribute to its pharmacological effect.

## Figures and Tables

**Table 1:** Serum biochemistry data at necropsy for all experimental groups of rats.

	TP	ALB	BUN	CREA	AST	ALT	ALP	Bil	TG	TCHO
Sham	5.4 ± 0.1	3.3 ± 0.1	20.4 ± 0.5	0.50 ± 0.05	123.1 ± 10.4	34.3 ± 5.8	485.5 ± 68.9	0.16 ± 0.03	114.7 ± 33.1	73.7 ± 4.4
VC	6.3 ± 0.9	4.1 ± 0.5	25.2 ± 4.3	0.56 ± 0.10	134.2 ± 22.5	33.7 ± 7.9	530.8 ± 110.3	0.17 ± 0.03	99.9 ± 33.6	86.6 ± 18.4
PC	6.5 ± 0.6	4.1 ± 0.3	25.0 ± 2.0	0.51 ± 0.05	132.4 ± 11.5	36.5 ± 5.5	595.8 ± 128.4	0.14 ± 0.04	130.7 ± 51.2	87.8 ± 21.8
T1	5.7 ± 0.1	3.8 ± 0.1	23.4 ± 2.6	0.52 ± 0.03	94.1 ± 8.7 *	31.9 ± 2.8	517.4 ± 103.3	0.17 ± 0.01	79.0 ± 26.9	78.4 ± 6.6
T2	5.7 ± 0.1	3.7 ± 0.1	21.4 ± 0.8	0.50 ± 0.09	100.6 ± 8.1 *	34.0 ± 3.3	435.3 ± 91.8	0.16 ± 0.03	113.3 ± 57.3	83.0 ± 9.4
T3	5.8 ± 0.2	3.8 ± 0.1	22.7 ± 2.2	0.52 ± 0.06	96.3 ± 8.0 *	31.6 ± 3.6	469.2 ± 91.9	0.16 ± 0.02	123.9 ± 59.3	74.8 ± 12.7
T4	5.6 ± 0.2	3.7 ± 0.2	20.1 ± 1.3	0.44 ± 0.03	82.5 ± 19.4 *	32.6 ± 2.1	358.8 ± 37.1*	0.18 ± 0.02	72.3 ± 19.9	73.8 ± 6.8

Serum levels of total protein (TP), albumin (ALB), blood urea nitrogen (BUN), creatinine (CREA), aspartate transaminase (AST) alanine transaminase (ALT), alkaline phosphatase (ALP) total bilirubin (Bil), triglyceride (TG), and total cholesterol (TCHO) was indicated as the mean value (± SD) of animals in each group. * *p* < 0.05 vs. the VC group. Sham, non-BPH-induced and received 0.5% CMC-Na; VC, BPH-induced and received 0.5% CMC-Na; PC, BPH-induced and saw palmetto-treated group.

**Table 2:** Relative composition of phenolic compounds in four *A. melanocarpa* extracts (T1~T4) as determined by LC/MS.

	Cyn-Hex	Cyn-Ara	Cyn-Pent	Cyn	CQ1	CQ2	QV
	(× 10^3^)						
T1	586	158	2.09	47.7	3.07	5.73	13.2
T2	268	68	1.08	20.9	2.21	3.55	10
T3	310	80.9	1.13	25.7	2.76	4.24	10.9
T4	230	56.8	0.69	18.4	2.45	3.86	0.88

T1, 100% ethanol/low temperature (30 °C) extract; T2, 100% ethanol/high temperature (100 °C) extract; T3, 60% ethanol/low temperature (30 °C) extract; T4, 60% ethanol/high temperature (100 °C) extract; Cyn-Hex: cyanidin-hexose, Cyn-Ara, cyanidin-3-arabinose; Cyn-Pen, cyanidin-3-pentose, Cyn, cyanidin, CQ1, caffeonylquinic acid; CQ2, caffeonylquinic acid; QV, quercetin vicianoside.

**Table 3:** Content of phenolic compounds in four *A. melanocarpa* extracts (T1~T4) as determined by HPLC.

Sample	Contents (mg/mL)			
	1-(3, 4-Dihydroxycinnamoyl Cyclopenta-2, 3-dilo)	Methyl 3-O-Caffeoylquinic Acid	Cyanidin-3-Glucoside	Cyanidin-3-Xyloside
T1	0.5349	ND	0.7642	2.6843
T2	0.5461	ND	0.5107	2.3228
T3	0.5015	ND	0.4559	2.1206
T4	0.5583	ND	0.6002	2.4279

T1, 100% ethanol/low temperature (30 °C) extract; T2, 100% ethanol/high temperature (100 °C) extract; T3, 60% ethanol/low temperature (30 °C) extract; T4, 60% ethanol/high temperature (100 °C) extract, ND: not detected.
